# Exploratory analysis of electronic patient-reported outcomes collection: comparing online and in-clinic modalities in cancer care

**DOI:** 10.1007/s11136-025-03975-2

**Published:** 2025-04-16

**Authors:** Eric Adjei Boakye, Mrudula Nair, Nada Al-Antary, Carl Wilson, Katelyn Kerr, Theresa M. Zatirka, Kelly A. Hirko, Farah Elsiss, Steven S. Chang, Benjamin Movsas, Michael Ryan, Samantha Tam

**Affiliations:** 1 Department of Public Health Sciences, Henry Ford Health, Detroit, MI USA; 2 Department of Otolaryngology-Head and Neck Surgery, Henry Ford Health, Detroit, MI USA; 3 Henry Ford Health + Michigan State University Health Sciences, Detroit, MI USA; 4 Department of Epidemiology and Biostatistics, Michigan State University College of Human Medicine, East Lansing, MI USA; 5 Wayne State University School of Medicine, Detroit, MI USA; 6 Henry Ford Cancer, Henry Ford Health, Detroit, MI USA; 7 Center for Health Policy & Health Services Research, Henry Ford Health, Detroit, MI USA; 8 Department of Radiation Oncology, Henry Ford Health, Detroit, MI USA; 9 Department of Public Health Sciences, Henry Ford Health, One Ford Place, Detroit, MI 48202 USA

**Keywords:** Patient reported outcome measures (PROMs), Patient reported outcome (PROs), Completion modality, Racial disparities, Online health platform, Cancer patients

## Abstract

**Purpose:**

Patient reported outcome measures (PROMs) have been shown to improve cancer survival but are generally underutilized in cancer care. It is unclear whether electronic-PROMS (ePROMs) modality (online vs. in-clinic) may address barriers to completion. We examined whether patient sociodemographic and clinical factors differed by completion modality.

**Methods:**

Patients with cancer who had an oncologic provider visit from January 2021 to March 2023 at a tertiary cancer center were assigned the National Institute of Health’s computer adaptive technology Patient-Reported Outcomes Measurement Information System instruments. Patients completed ePROMs either through online patient portal (online) up to 7 days before the visit or used a tablet at the clinic visit (in-clinic) if not completed online. Multivariable logistic regression model estimated associations between patient sociodemographic and clinical factors and completion modality.

**Results:**

A total of 8556 patients completed ePROMs (43.3% completed in-clinic). Females were less likely than males to complete ePROMs in-clinic (aOR = 0.89, 0.84–0.93) as were patients with commercial insurance (aOR = 0.83, 0.77–0.89) vs. Medicare; or saw radiation oncologist (aOR = 0.89, 0.83–0.96) vs. medical oncologist. However, patients were more likely to complete ePROMs in-clinic if they identified as Black race (aOR = 1.41, 1.33–1.49) vs. White; were single (aOR = 1.21, 1.14–1.29) or divorced/separated/widowed (aOR = 1.11, 1.04–1.18) vs. married; or saw a provider located in rural (aOR = 1.33, 1.25–1.42) vs. urban area.

**Conclusions:**

Patients who were males, Blacks, unmarried, Medicare insured or saw providers located in rural area were more likely to complete ePROMs in-clinic. Given the preference for online completion before visits for real-time symptom monitoring, targeted efforts are needed to boost online PROMs completion.

**Plain message:**

This is a cross-sectional analysis of the associations between sociodemographic and clinical factors with two electronic patient reported outcome measures completion modalities. The results indicate that about half of patients completed online and half completed in-clinic, with males, Blacks, patients who were divorced/separated/widowed, had Medicare insurance and saw a medical oncologist completing electronic patient reported outcome measures in-clinic. We support offering both options while addressing barriers to either modality.

## Introduction

Patient reported outcome measures (PROMs) are evidence-based patient communication tools that have begun to gain popularity and implementation across several fields of medicine, especially in cancer care [1]. PROMs help better incorporate patients’ voices into their own health care and provide their insight as an aid to clinical-decision making [2]. Some of the most prominent benefits of the information collected via PROMs are increased patient-provider collaboration, improved quality of life, reduced acute care utilization, and extended survival in cancer patients [1, 3, 4]. Additionally, PROMs create a space for dialogue between not only patients and clinicians but caregivers as well [5, 6]. Despite the benefits offered by PROMs to patients and providers, integration into routine practice remains limited. Moreover, further research is needed to standardize and optimize PROMs implementation throughout the healthcare system.

There are multiple barriers to the real-world implementation of PROMs. The most important barrier, in addition to time and workload barriers, is the low completion rate [7, 8]. Different factors can affect patients’ completion of PROMs, including technological and health literacy, language and cultural barriers, age, socioeconomic status, health status, race, and lack of interest or understanding of the questions, among others [9, 10]. As one of the main purposes of this instrument is to facilitate patient-centered medicine, patients’ adoption and motivation towards completing PROMs can play a major role in the success and efficacy of this tool in addition to securing buy-in from providers to employ this tool into their practice. In addition, unequal uptake of PROMs can exacerbate existing health care disparities. Therefore, a time- and cost-effective PROMs collection modality is needed to allow the successful integration of PROMs into standard of care.

PROMs can be administered in many formats, including mailed or in-person paper surveys, or digital surveys via tablets in clinical settings or at-home using online patient portals. Several studies have shown that administering PROMs in a digital format (ePROMs) is not only cost-effective and reliable but also improves completion rates through automated reminders and a streamlined workflow. Additionally, ePROMs help reduce administrative burden and minimize errors [11–13]. Moreover, the ease of incorporating ePROMs into Electronic Health Records (EHR) as opposed to a paper version could be vital for enhancing clinical care, boosting patient engagement, and overall improving health outcomes. It facilitates real time symptom monitoring with automated data collection and processing which can trigger alerts to healthcare providers when patients report severe or concerning symptoms. This enables early detection of complications, prompt intervention, and may help reduce symptom severity, complications, and hospital readmissions. Furthermore, there is currently a lack of literature on the best PROMs modality to address low completion rates by patients. Therefore, further investigation is needed regarding the optimal delivery method of PROMs in routine clinical settings. Our study aims to evaluate ePROMs completion modalities (using online portal or tablet provided in clinic) by patients at a tertiary cancer center and to evaluate the sociodemographic and clinical factors associated with each modality.

## Methods

### Data source and study sample

For this cross-sectional study, data from an implemented routine clinical ePROMs program at Henry Ford Cancer (HFC) within Henry Ford Health (HFH), a tertiary care hospital in southeastern Michigan, was utilized. In 2020, HFC implemented a patient reported outcomes program within the entire cancer service line. A committee comprising diverse HFC specialties (surgical/radiation/hematology oncology, neuro-oncology, supportive oncology, palliative medicine, public health sciences, nurses, quality, and cancer research) was created. The committee standardizes the PROMs collection process for HFC patients while balancing survey burden and maximizing value of patient feedback during cancer treatment. Providers were trained about the PROMs program and how to view results in the EHR among others. Four domains are evaluated using the Patient-Reported Outcomes Measurement Information System (PROMIS) computer adaptive testing (CAT): PROMIS^®^ CAT v2.0– Physical Function [14], PROMIS^®^ CAT v1.1– Pain Interference [15], PROMIS^®^ CAT v1.0– Depression [16, 17] and PROMIS^®^ CAT v1.0– Fatigue [18]. All adults (≥ 18 years old) with a previous diagnosis of cancer from any disease site with an oncologic visit are eligible for PROMs assessments, and the results are integrated within the Epic health record system. During active treatment (time in cancer trajectory in the proposed project), PROMs are offered bi-weekly for pain interference, physical function, and fatigue, monthly for depression. The questionnaires (offered only in English) are first offered online through MyChart^®^ patient portal (online modality group) 7 days prior to scheduled provider visit. At the time of the appointment check in, if the portal assessment was not completed, patients are offered a tablet to use to complete the PROMs in the waiting area (in-clinic modality group). Responses are uploaded to Epic immediately and available for review by the clinicians at the time of the visit. In the current workflow, patients with scores indicative of severe symptoms (two SDs worse than the US general population) are automatically referred to an urgent oncology clinic for triage or a social worker for further assessment. Oncology advanced practice providers and nurses from the urgent oncology clinic contact the person to offer the appropriate services, based on clinician decision-making [19]. The results are also available in the patient portal for patients to access. ePROMs scores are normalized with a mean T score of 50 points and standard deviation of 10 points. In this study, we restricted the analysis to first-time use of ePROMs between January 2021 and March 2023. HFH Institutional Review Board approved the study.

### Measures

The outcome variable extracted from EHR was completion modality, defined as the method used by patients to complete ePROMs, and classified as online (if patients completed PROMs via MyChart patient portal) or in-clinic (if patients completed PROMs via tablets offered in the clinic if the patient did not complete online). The independent variables included the following patient sociodemographic and clinical factors; age (continuous variable measured in years), self-reported sex (female, male), self-reported race (White, Black, Other [including all other races combined due to small counts]), self-reported marital status (married/significant other, divorced/separated/widowed, single), insurance type (commercial, Medicare, Other), phase of care– time in cancer continuum that a patient completed ePROMs– (pre-treatment [from diagnosis to onset of treatment], treatment [from onset of treatment to 6 months], post-treatment [6 months after treatment]), provider location– location where the patient’s oncology provider was located (urban, suburban, rural) and oncology provider specialty– the specialty the patient was seeing when they completed the ePROMs (medical, radiation, surgical, supportive).

### Statistical analysis

Descriptive statistics were used to describe the study population. Sociodemographic, clinical, and provider characteristics were compared between the online and in-clinic completion modality groups. Continuous variables were compared via Wilcoxon Ranked-Sum test (as the assumptions for a t-test were violated) and summarized via means and standard deviations as well as medians and interquartile (Q1, Q3) ranges. Categorical variables were compared via Chi-Squared test of Independence and summarized with frequencies and row percentages. A multivariable modified Poisson regression model was utilized to estimate associations between the patients’ sociodemographic, clinical, and provider characteristics (age, sex, race, marital status, insurance type, phase of care, provider location, and oncology specialty), and the outcome– ePROMs completion modality. Since the prevalence of the outcome was high (i.e., greater than 25%), modified Poisson regression model performs better at estimating risk compared to a binary logistic regression model. The model predicted the risk of completing ePROMs in-clinic vs. online. Statistical tests were 2-tailed, and 95% CIs not including 1 or *P* < 0.05 were considered statistically significant. All statistical analyses were conducted using SAS, version 9.4 software (SAS Institute Inc).

## Results

### Characteristics of study sample

Table 1 shows the characteristics of the study sample, overall and stratified by completion modality groups. Approximately 19,000 patients were offered the opportunity to complete the PROMs in the study timeframe. Out of the 19,000 patients offered, 8556 patients completed the PROMs yielding a completion rate of 45%. A total of 8556 patients were included in the study, of whom 3706 (43.3%) completed PROMs in-clinic (i.e., using tablet provided in clinic). The mean age of included patients was 64.8 (SD = 12.4) with a similar age distribution in both online and in-clinic groups. Most of the study population were females (64.5%, *n* = 5517), in which 57.7% (*n* = 3181) completed ePROMs online and 42.3% (*n* = 3706) completed ePROMs in-clinic (*P* = 0.0144). White race constituted 70.8% (*n* = 5841) of the total patient population; 61.5% (*n* = 3593) completed ePROMs online and 38.5% (*n* = 2248) completed ePROMs in-clinic whiles 43.4% (*n* = 865) of patients identifying as blacks completed ePROMs online and 56.6% (*n* = 1129) completed ePROMs in-clinic (*P* < 0.0001). Approximately 59% (*n* = 4980) patients were married or had a significant other (61.1% in the online group and 38.9% in the in-clinic group vs. 54.2% in the online group and 45.8% in the in-clinic group among patients who were divorced or separated vs. 46.5% in the online group and 53.5% in the in-clinic group among patients who were single (*P* < 0.0001)). Most patients, 59.3% (*n* = 5074) reported having Medicare insurance (54.3% completed PROMs online and 45.7% in-clinic whereas among patients who had commercial insurance, 64.0% completed ePROMs online and 36.0% in-clinic (*P* < 0.0001)).

**Table 1:** Characteristics of patients diagnosed with cancer who completed proms questionnaires at a tertiary cancer center (*N* = 8556)

	PROMs completion modality			
	Total^3^ (*N* = 8556)	Online^4^ (*N* = 4850)	In-Clinic^4^ (*N* = 3706)	*P*-value
**Age**				0.0809^1^
Mean (SD)	64.8 (12.42)	64.7 (12.26)	65.1 (12.63)	
Median (IQR)	66.0 (58.0, 73.0)	66.0 (57.0, 73.0)	67.0 (58.0, 74.0)	
**Sex**, n (%)				**0.0144** ^**2**^
Male	3039 (35.5%)	1669 (54.9%)	1370 (45.1%)	
Female	5517 (64.5%)	3181 (57.7%)	2336 (42.3%)	
**Race**, n (%)				**< 0.0001** ^**2**^
White	5841 (70.8%)	3593 (61.5%)	2248 (38.5%)	
Black	1994 (24.2%)	865 (43.4%)	1129 (56.6%)	
Other	416 (5.0%)	239 (57.5%)	177 (42.5%)	
Missing	305	153	152	
**Marital Status**, n (%)				**< 0.0001** ^**2**^
Married/Significant Other	4980 (59.0%)	3043 (61.1%)	1937 (38.9%)	
Divorced/Separated	1852 (21.9%)	1003 (54.2%)	849 (45.8%)	
Single	1613 (19.1%)	750 (46.5%)	863 (53.5%)	
Missing	111	54	57	
**Insurance Type**, n (%)				**< 0.0001** ^**2**^
Medicare	5074 (59.3%)	2754 (54.3%)	2320 (45.7%)	
Other	665 (7.8%)	294 (44.2%)	371 (55.8%)	
Commercial	2817 (32.9%)	1802 (64.0%)	1015 (36.0%)	
**Phase of Care**, n (%)				**< 0.0001** ^**2**^
Post-Treatment	5089 (59.5%)	2784 (54.7%)	2305 (45.3%)	
Treatment	2534 (29.6%)	1505 (59.4%)	1029 (40.6%)	
Pre-Treatment	933 (10.9%)	561 (60.1%)	372 (39.9%)	
**Provider Location**, n (%)				**< 0.0001** ^**2**^
Urban	4721 (55.2%)	2531 (53.6%)	2190 (46.4%)	
Rural	1681 (19.6%)	809 (48.1%)	872 (51.9%)	
Suburban	2154 (25.2%)	1510 (70.1%)	644 (29.9%)	
**Oncology Specialty**, n (%)				**< 0.0001** ^**2**^
Supportive	167 (2.0%)	98 (58.7%)	69 (41.3%)	
Radiation	1463 (17.1%)	887 (60.6%)	576 (39.4%)	
Surgical	1224 (14.3%)	650 (53.1%)	574 (46.9%)	
Medical	4929 (57.6%)	2699 (54.8%)	2230 (45.2%)	
Unknown	773 (9.0%)	516 (66.8%)	257 (33.2%)	

^1^Kruskal-Wallis p-value; ^2^Chi-Square p-value; PROMs = Patient-report outcome measures; ^3^Column percents are presented; ^4^Row percents are presented

Most of the of patients, 59.5% (*n* = 5089) were in the post-treatment phase of whom 54.7% (*n* = 2784) completed ePROMs online and 45.3% (*n* = 2305) completed ePROMs in-clinic whilst 60.1% (*n* = 561) of those in pre-treatment phase completed ePROMs online and 39.9% (*n* = 372) completed ePROMs in-clinic (*P* < 0.0001). Approximately 55% (*n* = 4721) of patients saw a provider located in an urban location, while 25.2% (*n* = 2154) and 19.6% (*n* = 1681) saw a provider located in suburban and rural locations, respectively. Among patients who saw a provider located in urban areas, 53.6% (*n* = 2531) completed ePROMs online and 46.4% (*n* = 2190) in-clinic and among those that saw a provider located in suburban areas, 70.1% (*n* = 1510) completed ePROMs online and 29.9% (*n* = 644) in-clinic (*P* < 0.0001). Most patients had a visit with a medical oncology specialist (63.3%, *n* = 4929) followed by a visit with radiation oncology specialist (17.1%, *n* = 1463) and surgical oncology specialist (13.3%, *n* = 1224).

### Factors associated with completion modality

**Fig. 1:**
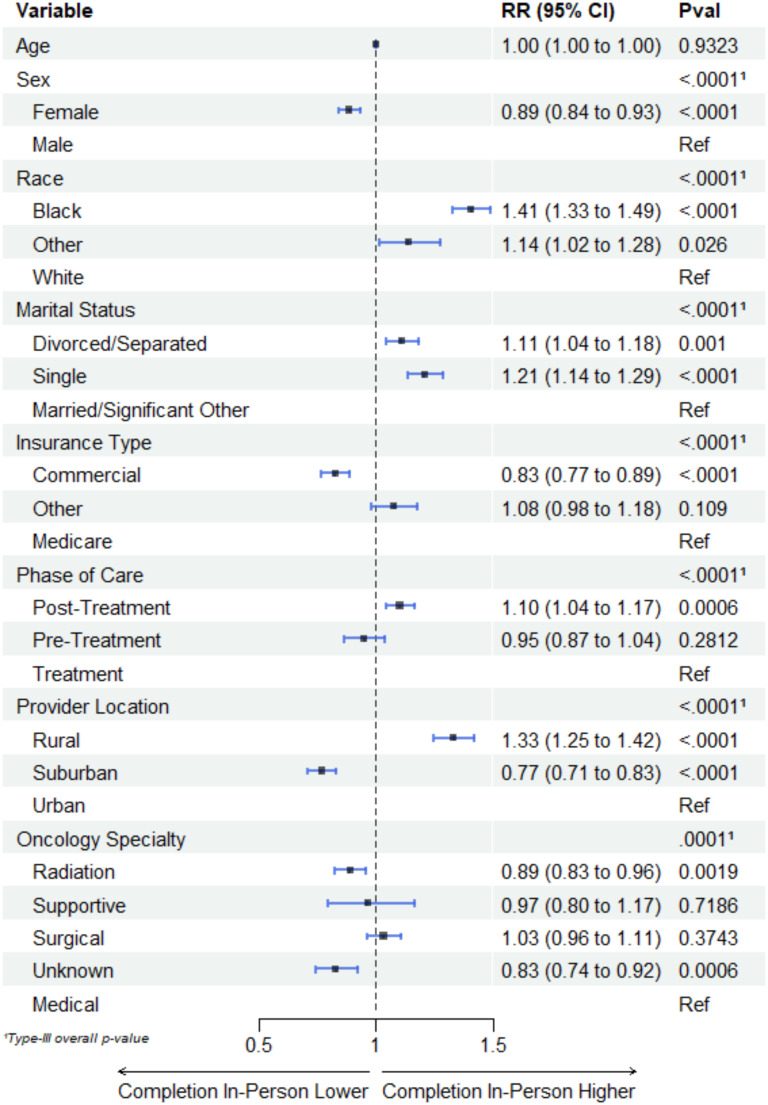
Factors associated with in-clinic PROMs completion at a tertiary cancer center (*N* = 8556)

As shown in Fig. 1, compared with male patients, female patients were 11% less likely to complete ePROMs in-clinic (aRR = 0.89, 0.84–0.93). Patients identifying as Black had a 41% higher risk (aRR = 1.41, 1.33–1.49) or as Other races had a 14% higher risk (aRR = 1.14, 1.04–1.18) of completing ePROMs in-clinic compared to those identifying as White. Similarly, compared to patients who were married or had a significant other, patients who were single had a 21% higher risk (aRR = 1.21, 1.14–1.29) or divorced/separated/widowed had 11% higher risk (aRR = 1.11, 1.04–1.18) of completing ePROMs in-clinic. Patients in the post-treatment phase had a 10% higher risk (aRR = 1.10, 1.04–1.17) of completing ePROMs in-clinic compared to those in the treatment phase. On the other hand, compared to patients who had Medicare insurance, those who had commercial insurance were 17% were less likely (aRR = 0.83, 0.77–0.89) to complete ePROMs in-clinic. Compared to patients who received care from a provider located at an urban location, patients who received care from a provider located at a rural location were 33% more likely (aRR = 1.33, 1.25–1.42) to complete ePROMs in-clinic, whereas those who received care from aprovider at a suburban location 23% less likely (aRR = 0.77, 0.71–0.83) to complete ePROMs in-clinic. Finally, patients who saw a radiation oncology specialtist were 11% less likely (aRR = 0.89, 0.83–0.96) to complete ePROMs in-clinic compared to those who saw a medical oncology specialist.

## Discussion

This study explored the associations between sociodemographic-, clinical-, and provider-level factors and two common ePROMs completion modalities (online vs. in-clinic) in a tertiary cancer center. It should be noted that the study focuses on two digital completion modalities rather than digital vs. in-clinic paper and pencil modality. Previous studies examined various PROMs completion modalities, with a focus on paper-based formats [20–23]. To the best of our knowledge, this is the first study comparing electronic-based PROMs completion modalities integrated into routine cancer care. Our results suggest that both online and in-clinic modalities were frequently utilized by patients with cancer, with a slight preference (56.7%) towards online PROMs completion. Given the nearly equal utilization of both modalities by patients, both options should be kept available according to patient preference while addressing barriers withholding patients from using either method.

Even though both modalities are electronic-based approaches to completing PROMs, completion online prior to the scheduled clinic visit is considered good practice for real-time symptom monitoring [24–26]. This could provide maximal benefit to patients, as completing the questionnaire online ensures timely interventions as required, especially if patients report severe scores for any of the assessed domains [27]. Moreover, it is possible that patients may not get sufficient time to complete the questionnaires in the clinic and they could be rushed or may not have the time to complete the questionnaire entirely resulting in partially completed questionnaires. In-person settings, such as clinics or hospitals, may be more stressful due to time constraints, interactions with healthcare providers, or the clinical atmosphere itself. This could result in the patients not being fully evaluated prior to the appointment and increasing the possibility that their issues may be unrecognized and unaddressed. Additionally, medical support staff who provide PROMs on-site assistance might face multi-level challenges related to the workload and operational burden [28]. Medical support staff are also asked to use their discretion as to how willing or able patients are to complete administrative tasks when they arrive in clinic. For instance, a previous study reported that, patients who appeared visibly unwell were not offered the survey tablet by medical support staff [28]. Despite the benefits of online completion prior to the clinic visit, it may be an unfeasible option for some patients including those who prefer in-person completion or lack internet and technology access. Thus, in-clinic completion should still be made available to patients, with efforts directed to tackle the barriers that are limiting completion.

Our findings suggest that Black patients were more likely than White patients to complete ePROMs in-clinic. Similarily, patients who saw providers at rural locations were more likely to complete ePROMs in-clinic compared to those who saw providers at urban locations. Several factors could explain these findings. One possible explanation is the limited access to internet, smartphone and other modes of internet connection among Black patients compared to other racial and ethnic populations, which had been documented in previous studies [29, 30]. Limited access to broadband and computers [31] may also explain why patients receiving care at rural areas are more likely to complete ePROMs in-clinic as compared to those at surburban and urban areas. Another potential explanation for these findings could be related to the lower utilization of the online patient portal as a whole by Black patients. For instance, a study found that African American patients had a lower likelihood of activating a patient portal compared with White patients [32]. In addition, according to a study [33] conducted by Kaiser Permanente- Northern California, patients who were white, female and aged between 30 and 65 had higher chances of registering for the online healthcare portal compared to others. Therefore, it is possible that, individuals with low completion rates who are experiencing deteriorating health may wait until their next scheduled visit to report their symptoms in-person, or alternatively, seek immediate attention through emergency room [34, 35]. In contrast, by reporting symptoms through ePROMs, these patients could benefit from prompt care and supportive oncology services [6, 34]. Therefore, additional strategies are needed to effectively address barriers keeping patients from using online modalities for ePROMs completion in order to avoid disparities in clinical care.

In this study, those who were single or divorced/separated were more likely to complete ePROMs in-clinic compared to married patients. This finding is consistent with a previous study, in which single or divorced/separated individuals exhibited lower rates of PROMs completion and digital health literacy [36]^,^ [37–39] compared to their married counterparts. Digital literacy limitations could explain why single and divorced/separated patients are less likely to complete PROMs online before visits. Studies often attribute this disparity to the hypothesis that married individuals prioritize their health and benefit from stronger social support [40], factors that could facilitate higher engagement with digital health activities and frequent encouragement to report their symptoms remotely.

We found that patients who visited radiation oncology specialists were less likely to complete ePROMs in-clinic compared to those visiting medical oncology specialists in our study population. This discrepancy could be due to the nature of patient-provider relationships. Patients tend to build an interactive rapport [41, 42] with their medical oncology team mainly due to the increased frequency of in-clinic clinic visits compared to other oncology specialists. Additionally, the broad range of symptoms addressed by medical oncology creates a space where topics covered in PROMs surveys are more likely to be discussed during consultations. This familiarilty and frequent interaction could contribute to why some patients are more likely to complete PROMs at check-in as they are more familiar with the staff, nurses, and medical assistants, rather than opting for online completion. We also found that patients who were in the post-treatment phase were more likely to complete ePROMs in-clinic compared to those in the treatment phase. This could be because during the post-treatment phase, patients may not check online portal as much as during treatment phase, thus they may not see the prompt in patient portal to complete it. Also, it is possible patients in the post-treatment phase are less focused on their disease and busy with doing other things which make them less likely to see messages to complete the PROMs in patient portal.

Several factors could influence ePROMs completion modality selection including limited digital and health literacy, general engagement with the healthcare system, access to internet, and physical impedence such as visual impairment or dexterity issues. Therefore, a combination of both methods (online and in-clinic) may be most effective to promote ePROMS completion and maximize on potential benefits. Although the online option is the preferred method for completing ePROMs, ensuring inclusivity is vital. Providing patients with alternative completion options is crucial for enhancing completion rates and minimizing disparities. Further evaluation is necessary to determine patient preferences for completing the questionnaire in-clinic as opposed to online, in order to identify barriers to online completion and develop interventions to improve ePROMs completion rates. Further research is also warranted to explore alternative completion modalities for ePROMs, such as Interactive Voice Response (IVR). IVR utilizes automated telephone technology that permits callers to interact with voice response system through speech recognition or a keypad. This could be particularly beneficial for those facing challenges with technology access, physical impairments that affect screen use, internet connectivity issues, and other barriers [6]. Offering PROMs in languages other than English could also improve completion rates among non-English speaking patients.

### Strengths and limitations

A strength of the study is the large sample size. The study analyzes data from 8,556 patients, which provides a substantial sample size for robust statistical analysis and increases the reliability of the findings. The study has limitations. First, the findings may not be generalizable nationally since the data only reflects one local health care system. Second, data regarding online MyChart patient portal enrollment and health literacy assessment were not available, thus we were not able to adjust for their effects on completion modality. Third, we do not have exact data on patients who were not offered the opportunity to complete ePROMs. However, we believe this will not be a large number of patients as there are several opportunities for cancer diagnosis to be documented. Fourth, there is lack of information on disease stage. Patients with more advanced disease may be harder to reach for ePROM collection. Finally, providers were not asked about their preferred modes of communication with patients, so if providers in one oncology specialty do not utilize MyChart to communicate with patients online as often as providers in a different specialty, rates of online PROMs completion could have been affected. Additionally, this limitation extends to patient preferences regarding online versus in-clinic completion, particularly for those who may face barriers in accessing the online portal at home. Future studies should explore both provider and patient perspectives to optimize ePROM collection methods.

## Conclusions

PROMs are valuable tools in improving cancer care; however, further efforts are necessary to effectively incorporate PROMs into cancer routine practice and increase their utilization. Our research indicates that patients of Black race, males, those who are single or divorced/separated, have Medicare insurance, receive care from providers located in rural locations or from medical oncology specialty are more likely to complete PROMs questionnaires in-clinic as opposed to online. As a result of our findings as well as those of previous studies, we advocate for offering both in-clinic and online options for completing PROMs while tackling the multi-level barriers that are keeping patients from using either modality. Providing multiple modalities for completion can lead to increases in completion rates, thereby improving access to PROMs which could support delivery of care by promptly addressing patients’ symptoms and offering supportive services when needed.

## Declarations

### Prior meeting presentation

The abstract of the manuscript was presented as a poster presentation at the 2023 ASCO Annual Meeting, Jun 2–6, 2023; Chicago, IL, USA.
